# Surgical reconstruction of the external nose: Alar rotational and hinged flap techniques for facial cleft Tessier 0–1 with lipoma on dorsal nasal region - A case report

**DOI:** 10.1016/j.ijscr.2024.109708

**Published:** 2024-04-26

**Authors:** Hardisiswo Soedjana, Lisa Y. Hasibuan, Arif Tri Prasetyo, Kevin Leonard Suryadinata

**Affiliations:** Division of Plastic Reconstructive and Aesthetic Surgery, Department of Surgery, Faculty of Medicine, Universitas Padjadjaran, Bandung, Indonesia

**Keywords:** Case report, Facial cleft, Tessier, Cleft nose, Alar transpositional flap, Hinge flap

## Abstract

**Introduction and importance:**

Tessier Craniofacial Clefts Numbers 0 and 1 represent unique facial deformities, with Number 0 involving midline structure hypoplasia and Number 1 exhibiting features like a notched soft triangle and affected alar dome. These anomalies can extend near the midline, leading to complications like telecanthus, necessitating innovative surgical strategies for reconstruction.

**Case presentation:**

A five-month-old girl presented with Tessier 0 and 1 clefts and a dorsal nasal lipoma, challenging traditional repair methods due to structural limitations. This case required a comprehensive approach, including aesthetic excision of the lipoma and reconstruction of both the internal and external aspects of the nose.

**Clinical discussion:**

The patient underwent successful nasal reconstruction using a transpositional alar flap with a pedicle from the angular artery and a hinge flap for the inner lining. The procedure involved cranial dissection through the flap incision for lipoma excision. This case highlights the complexity of nasal reconstruction in the presence of facial clefts and demonstrates the effectiveness of the alar transpositional flap as a viable technique for achieving aesthetically pleasing outcomes.

**Conclusion:**

The case underscores the necessity for precise surgical planning and execution to address both cosmetic and functional aspects of nasal defects in facial cleft patients.

## Highlights

• Unique challenge: Tessier 0 and 1 clefts with dorsal nasal lipoma complicate reconstruction
• Alar and hinge techniques: employed for effective aesthetic nasal repair
• Tailored surgical strategy: addresses both internal and external nasal defects

## 1 Introduction

Craniofacial abnormalities are predominantly recognized through their physical manifestations. The classifications of these malformations are generally based on clinical or anatomical criteria, often overlooking the malformation's developmental stage and the underlying pathology. With an occurrence rate of approximately 1 in 10,000,000 births, these malformations can appear sporadically or as part of a genetic sequence of anomalies. Embryologically, they result from the incomplete merging of the medial nasal prominences [1,2].

Craniofacial clefts and orbital hypertelorism (OHT) are two separate conditions that share a wide range of presentation intensities. These can vary from minor irregularities with negligible clinical impact to some of the most severe cases of craniofacial deformity observed in plastic surgery. Except for a few instances, such conditions are uncommon and typically arise from undefined disturbances in embryogenesis. Nevertheless, there are several established syndromes and conditions primarily characterized by hypertelorism or craniofacial clefting [3]. Collectively, it's more accurate to consider these conditions as part of a continuum of phenotypic expressions rather than entities with a uniform cause and development process. Therefore, constructing an inclusive approach to the comprehension, assessment, and management of individuals afflicted with these intricate disorders is essential for effective treatment [4].

Various techniques exist for closing nasal defects to achieve both aesthetic balance and functional integrity, including the use of local regional flaps. A deep understanding of local flap designs is crucial for repairing alar defects to minimize the risk of distortion and potential complications. While the melolabial flap remains a primary method for addressing nasal defect areas, the local flap from the nose has been recognized as an alternative offering fewer restrictions compared to other local flaps [5]. This case report aims to document the application of the alar transpositional flap for treating nasal clefts in conjunction with Tessier 0 and 1 facial cleft who also got additional lipoma on the dorsal of the nose.

## 2 Presentation of case

A five-month-old girl was brought to the Plastic Surgery Outpatient clinic at our hospital due to a cleft on the left side of her nose. The left alar was notably asymmetric, tilting upward towards the cranial direction, and a mass was observed on the cranial side of the nose. Initially, we suspected it might be a meningoencephalocele. Consequently, a CT scan was conducted to determine the presence of any nasal bone defects. The CT scan revealed only a minor defect on the nose that would not allow brain tissue to protrude. Further ultrasound examination identified the mass as fatty tissue, leading to a diagnosis of a lipoma in the nasal area for our patient.

**Fig. 1:**
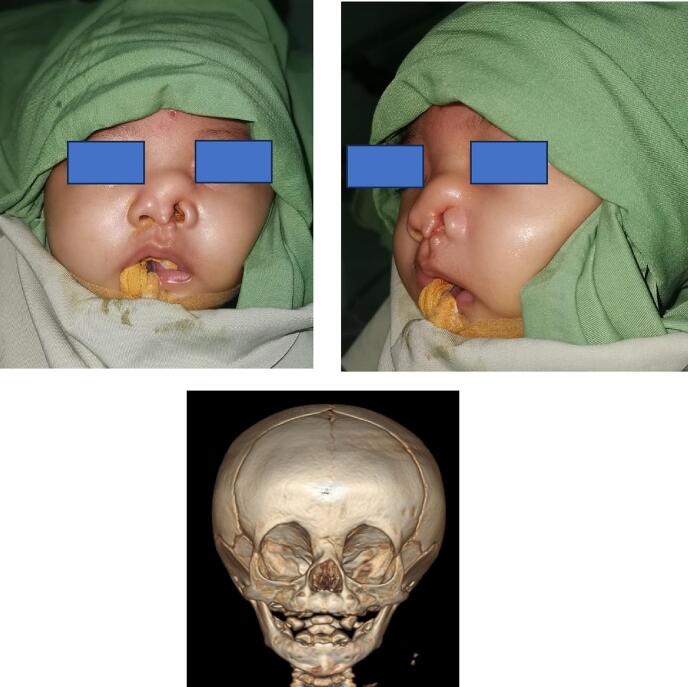
Clinical and radiological manifestation of the patient with left facial cleft Tessier 0–1 with lipoma on the dorsal of the nose.

As illustrated in Fig. 1, the patient exhibited multiple nasal abnormalities, such as malposition of the left alar, lack of the nasal inner lining, and the presence of a lipoma on the dorsal nasal region necessitating surgical correction. The objective of the surgery was to anatomically and aesthetically reconstruct the nose.

**Fig. 2:**
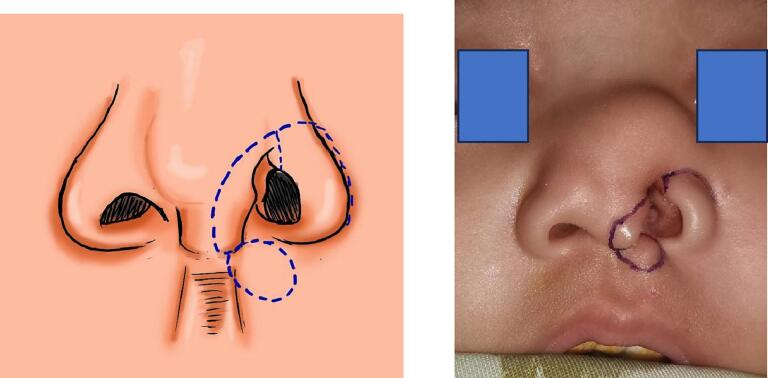
Incision design.

We devised a plan to utilize an alar rotational transpositional flap combined with a hinge flap to reconstruct the inner lining. From the incision made for the rotational flap, we proceeded with cranial dissection to excise the lipoma. The design of the operation is detailed in Fig. 2.

**Fig. 3:**
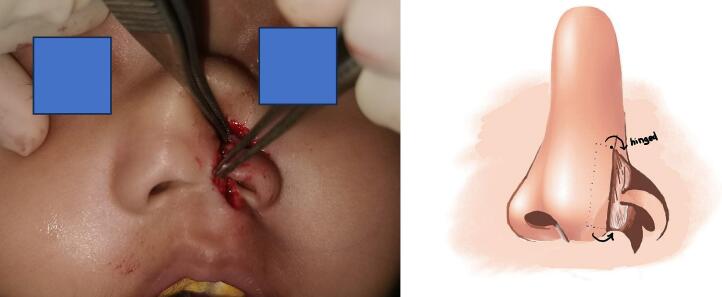
Illustration and condition during surgery.

**Fig. 4:**
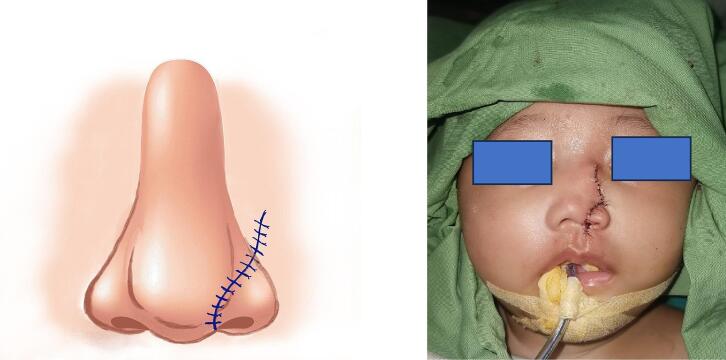
Immediate post surgery result.

In Fig. 3, following the planned incisions, we were able to construct the nasal inner lining using a hinge flap and perform the rotational flap. Fig. 4 presents a post-surgery image, displaying aesthetically pleasing results.

**Fig. 5:**
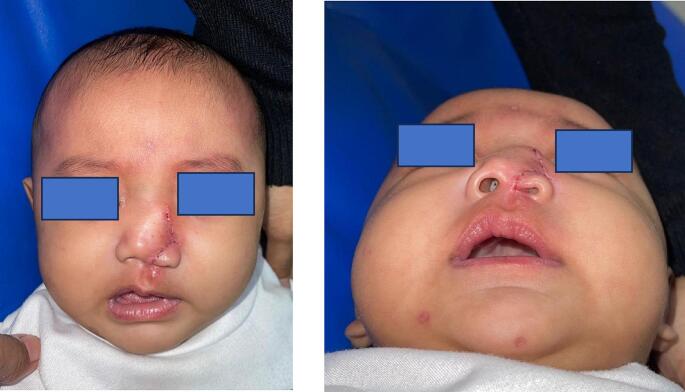
Clinical condition, a week after surgery.

The patient was discharged from the hospital the day after the surgery and returned to the outpatient clinic a week later for suture removal. The outcome was favourable, as depicted in Fig. 5. Moving forward, the patient will be continually evaluated and undergo further surgeries to refine the nasal shape as needed.

Before the procedure, the patient underwent the process of obtaining informed consent regarding the impending intervention. This step is crucial, especially for the abdominal flap procedure, as it involves the creation of a mass that necessitates full immobilization of the hand until the flap stabilizes. Additionally, the patient was also provided with informed consent regarding potential publication and this work has been reported in line with the SCARE criteria [6].

## 3 Discussion

Facial clefts Tessier 0–1 are characterized by anomalies along the midline that slightly extend laterally. In this patient, the defect was observed in the soft tissue along the lines indicative of facial clefts 0 and 1. According to the classification, there was a notable anomaly in the nose, clinically manifested by asymmetric alar wings [3,4].

Various methods are available for reconstructing the nose in patients with facial clefts, with the glabellar flap being one of the most commonly employed techniques. This method utilizes the glabella as an integral part of the nose, employing a V-Y incision to correct malpositioned nasal structures [7]. However, in this patient, a glabellar flap was not feasible due to a cranial defect from the cleft in the form of a lipoma, which would affect the vitality of the flap. Consequently, an alar flap was chosen for this case. Studies by Koch, C. et al. (2011) demonstrate the effectiveness of the glabellar flap in scenarios of nasal reconstruction, providing a contrast to our case where such a method was deemed inappropriate due to the presence of a lipoma compromising flap vitality. Conversely, Krishnamurty, A (2018) have documented the use of combined flap techniques in cases with extensive tissue damage, illustrating alternative approaches that, while informative, were too complex for the needs presented by our patient. These references underline the importance of tailoring flap choices to the specific anatomical and pathological conditions of each patient [8,9].

The hinge flap is a straightforward flap modality employed in this case to reconstruct the nasal inner lining. This technique is commonly utilized in nasal reconstruction cases involving oncology [10,11]. For patients with facial clefts, it can also be applied to achieve more optimal reconstruction outcomes.

Facial cleft 0 is often accompanied by other abnormalities, such as meningoencephalocele, especially when there is a significant defect in the nasal bone [12,13]. A large defect could allow brain tissue to protrude, hence in cases of facial cleft with a suspicion of meningoencephalocele, it is essential to conduct CT scan and, if necessary, MRI examinations to ascertain the extent of the defect. In this case, no significant defect was found in the nasal area, thus ruling out the suspicion of meningoencephalocele.

In this particular case, the decision to forego histopathological examination was made based on the distinctive clinical and ultrasonographic characteristics of the lesion, which strongly indicated a lipoma. The mass presented with all the classical attributes typically associated with lipomas—being soft, mobile, and well-circumscribed, coupled with its homogeneous hypoechoic appearance on ultrasound, which lacks any vascular features. These findings provided sufficient confidence in the diagnosis, allowing us to rely on clinical judgment and ultrasound alone, avoiding the need for histopathological confirmation. This approach aligns with our center's protocol for managing clear-cut cases, optimizing resource utilization without compromising the accuracy of the diagnosis.

## 4 Conclusion

Numerous treatment modalities exist for addressing a cleft nose resulting from facial clefts. It's crucial to evaluate all factors involved to ensure the nose is reconstructed in a manner that is both anatomically correct and aesthetically pleasing.

## Ethical approval

Ethical approval for this study (Ethical Committee N° NAC 207) was provided by the Ethical Committee of Dr. Hasan Sadikin General Hospital, Bandung, Indonesia on March 1^st^, 2024.

## Funding

This research did not receive any specific funding.

## Author contribution

Hardidiswo Soedjana: Study concept or design, data collection, data analysis, interpretation, writing the paper.

Lisa Y. Hasibuan: Study concept or design, data collection, data analysis, interpretation, writing the paper.

Arif Tri Prasetyo: Study concept or design, data collection, data analysis, interpretation, writing the paper.

Kevin Leonard Suryadinata: Study concept or design, data collection, data analysis, interpretation, writing the paper.

## Guarantor

Hardisiswo Soedjana

Lisa Y. Hasibuan

Arif Tri Prasetyo

Kevin Leonard Suryadinata

## Research registration number

This study does not require registration.

## Conflict of interest statement

The authors declare no conflicts of interest related to this study.
